# Association Between Depressive Symptoms and Cardiac Structure and Function in a Peruvian Population

**DOI:** 10.5334/gh.981

**Published:** 2022-10-27

**Authors:** Christine Santiago, Melissa Burroughs Peña, Timothy Brown, Saate Shakil, James Januzzi, Eric Velazquez, J. Jaime Miranda, Danny Rivera, William Checkley

**Affiliations:** 1 Stanford Department of Medicine, Palo Alto CA, USA; 2 School of Public Health, University of California, Berkeley, Berkeley, CA; 3 Division of Cardiology, Stanford Health Care, Oakland, CA, USA; 4 Division of Cardiology, University of Washington, Seattle, Washington, USA; 5 Division of Cardiology, Department of Medicine, Mass General Hospital, MA, USA; 6 Section of Cardiovascular Medicine, Department of Internal Medicine, Yale School of Medicine, New Haven, CT, USA; 7 CRONICAS Center of Excellence in Chronic Diseases, Universidad Peruana Cayetano Heredia, Lima, Peru; 8 Department of Medicine, Universidad Peruana Cayetano Heredia, Lima, Peru; 9 Division of Cardiology, Department of Medicine, Duke Clinical Research Institute, Duke University Medical Center, Durham, NC, USA; 10 Division of Pulmonary and Critical Care Medicine, School of Medicine, Johns Hopkins University, Baltimore, MD, USA; 11 Center for Global Non-Communicable Disease Research and Training, School of Medicine, Johns Hopkins University, Baltimore, MD, USA

**Keywords:** Echocardiography, Depression, Diastolic Function

## Abstract

Depressive disorders are a leading cause of disability and are globally pervasive. It is estimated that 80% of depression occurs in low-income and middle-income countries. Depression is associated with worse outcomes in patients with cardiac disease including heart failure (HF); however, mechanistic understanding to explain heightened risk in HF remains poorly characterized. We examined the association between depressive symptoms and cardiac structure and function by transthoracic echocardiography. We selected a random sample of adult participants in Puno and Pampas de San Juan de Miraflores, Peru, from the CRONICAS cohort study. Depression symptoms were self-reported and measured with the Center for Epidemiological Studies Depression Scale in 2010. Participants underwent transthoracic echocardiography in 2014. Multivariable linear regression was used to examine the relationship between depressive symptoms and echocardiographic measures of cardiac structure and function and was adjusted for relevant covariates. Three hundred and seventy-three participants (mean age 56.7 years, 57% female) were included in this analysis of which 91 participants (24%) had clinically significant depressive symptoms. After adjustment, clinically significant depressive symptoms were associated with a reduced diastolic relaxation velocity compared to non-depressed subjects (–0.72 cm/s, 95% CI –1.21 to –0.24, p = 0.004). Other differences between depressed and non- depressed participants were less obvious. In conclusion, clinically significant depressive symptoms were associated with a lower septal e’ velocity in the Peruvian population. Depressive symptoms were not obviously associated with other abnormalities in cardiac structure or function.

## Introduction

Mood disorders are a leading cause of disability worldwide with 4.4% of the world population suffering from depression [1]; in 2017, depressive disorders were the third leading cause of years living with disability globally for both sexes combined [2]. Beyond being prevalent, the incidence of depression is growing: the number of people around the world living with depressive disorders has increased by 18.4% from 2005 to 2015. Accordingly, depressive disorders comprise approximately 14% of the global burden of disease. Notably, depressive disorders have an unequal distribution globally, where 80% of the disease burden occurs in low- income and middle- income countries such as Peru [1]. For example, in Lima, Peru the estimated prevalence of depression is approximately 17% [3]. The unequal distribution is attributed to the growing population in low-income and middle-income countries living to the age where depressive disorders more commonly occur [1].

Of concern, depressive disorders are associated with higher risk for the development of coronary artery disease and incident myocardial infarction [4]. Studies have also shown a bi-directional relationship between heart failure and depression; on the one hand, those with heart failure have a considerably higher prevalence of depression, while on the other hand, those with depression are at a higher risk for incident heart failure. Relative to the latter finding, for example, among elderly subjects with isolated systolic hypertension there was a two times higher risk of developing heart failure in depressed versus non-depressed groups [5]. This association was also found in elderly type 2 diabetics in Australia [4]. Whether heightened risk for incident heart failure among depressed individuals is related to direct cardiac effects of depressive disorders is not clear; depression is associated with numerous physiologic effects that may increase risk for incident heart failure, including impact on hemodynamics, hematologic indices, and neurohormonal status [6]. Despite this fact, the effect of depression on cardiac structure and function in depressed versus non-depressed populations has not been extensively studied in the literature. Given the lack of large scale, prospective studies analyzing the effect of depression on heart failure, we evaluated sub-clinical changes in cardiac structure and function to provide more information about the physiologic changes that occur in response to depression. We sought to study the association between clinically significant depressive symptoms and both cardiac structure and function as measured by transthoracic echocardiography in two settings in Peru.

## Methods

The CRONICAS study was a longitudinal study started in September 2010 that took place in three different geographically distinct settings in Peru to better understand the risk factors for chronic disease [7]. We conducted surveys on a variety of validated measures such as depression, exposures and health risk factors. The study design was a simple random sample stratified by sex, age and geographic location, with the aim to recruit 1,000 participants at each of the three study sites, Puno, Tumbes and Lima. Participants in Puno were further stratified by rural and urban dwelling. Pampas de San Juan de Miraflores (Pampas) is a poor peri-urban community located 25 km south of Lima and consists of Andean immigrants [5]. Puno is a city located in southeastern Peru with both an urban and rural community.

This ancillary study was conducted from a simple random sample of CRONICAS participants in Puno and Lima. Due to funding and time constraints, Tumbes was not included, and a subset of participants were selected from the Puno and Lima sites. CRONICAS participants aged 35 years and older who were full time residents of Puno and Pampas site were selected for participation. The aim of the study was to recruit 400 participants, approximately 200 in Lima and approximately 200 in Puno. We utilized the baseline CRONICAS surveys collected in 2010, which included data on depression status, and echocardiogram data collected in 2014. Exclusion criteria for the CRONICAS protocol included individuals who were pregnant, unable to provide informed consent or respond to questionnaire and individuals with any physical limitations that prevented them from providing accurate measurements. Only one participant per household was enrolled in the study.

The baseline survey was completed in September 2010. Questionnaires were administered face-to-face with trained community health workers. They were conducted according to the World Health Organization STEP methodology and conducted in three languages, Spanish, Aymara and Quechua [7].

Participants received transthoracic echocardiography that was performed by either a senior sonographer or cardiology fellow using a portable ultrasound machine (M-Turbo, Sonosite/Fujifilm, Bothell, WA). The transthoracic echocardiogram protocol developed specifically for the study was adapted from the Duke Cardiac Diagnostic Unit echocardiography protocol (unpublished). 2D imaging was used to acquire cardiac views including the parasternal long axis, parasternal short- axis, apical four chamber, apical two- chamber, apical three chamber and subcostal views.

The echocardiography protocol used is identical to a previously published study except myocardial strain was not calculated in the present analysis [8]. The following echocardiogram variables were measured: left atrial area in four- chamber and two-chamber views, left atrial anterior- posterior diameter, left ventricular mass, left ventricular internal diameter in systole, left ventricular internal diameter in diastole, left ventricular ejection fraction, tissue doppler measure of early myocardial relaxation velocity (e’) at the lateral and septal positions, ratio of the mitral inflow peak E to A wave velocity (E/A ratio), ratio of the mitral inflow peak E wave velocity to the early myocardial relaxation velocity (E/e’), right ventricular width at the base and mid- cavity, right ventricular length, right ventricular systolic pressure (RVSP), tricuspid annular plane systolic excursion (TAPSE) and right ventricular outflow tract time to peak velocity. Left ventricular ejection fraction was calculated by Simpson’s biplane method, using the left ventricle in end- systole and end- diastole in the apical four- chamber and apical two- chamber views. The left atrial area was calculated from the left atrium in the apical four- chamber and two- chamber views. Maximum and minimum inferior vena cava diameter, one inch from the hepatic vein in the subcostal view was used to estimate right atrial pressure; RVSP was estimated from the tricuspid regurgitant jet velocity using the modified Bernoulli equation. Left ventricular mass was calculated using the American Society of Echocardiography Equation using the 2D parasternal long-axis images [9]. The echocardiography images were interpreted by a resident and a cardiologist blinded to the research participants exposure status or self- reported mental health responses.

The following variables were acquired from a self-reported structured questionnaire: pack-years of tobacco smoking, prior diagnosis of diabetes, daily biomass fuel use (previously linked to incident heart disease), physical activity, depression, and hazardous alcohol consumption. Significant depressive symptoms were defined as having a score on the Center for Epidemiological Studies Depression Scale (CES-D) of 24 or greater. The CES-D questionnaire has been validated in Spanish speaking countries and a score of 24 or greater in community dwelling populations has been estimated to have a sensitivity of 91.4% and specificity of 96.7% for detecting depression [10]. Hazardous alcohol utilization was determined by using the Alcohol Use Disorders Identification Test [11]. Physical activity was determined based on the International Physical Activity Questionnaire as recommended for Latin American populations [12,13]. Daily biomass utilization was determined by self- reported utilization of daily wood or dung for cooking for more than six months at a time during the participant’s lifetime.

The aim of this cross-sectional study was to assess cardiac structure and function of the right and left heart in participants with and without depressive symptoms. The baseline characteristics of symptomatically depressed and non- depressed participants was compared using chi- squared tests for categorical variables and t-tests for continuous variables. All echocardiographic outcome parameters were assessed individually for normality by using histograms, q-q plots and univariate kernel density estimations. Two parameters were log transformed because they were not normally distributed, this includes left ventricular mass and E/A ratio.

Limited multivariable and full multivariable regression analysis were used to model the association between depressive symptoms and heart structure and function to make sure there was no overfitting. The limited variable model adjusted for age, sex, site, exposure to biomass fuel smoke and height. The full model additionally adjusted for BMI, total physical activity, prior diabetes diagnosis, pack- years of smoking, hazardous alcohol consumption as well as systolic and diastolic blood pressure. Linear regression was used for all echocardiographic parameters, stratified by sex. All models were corrected for arbitrary forms of heteroscedasticity. The statistical analysis was conducted in STATA/MP 15.1 (Stata Corp, College Station, TX, USA).

Secondary analyses examined the depression variable from discrete to continuous (Appendix Table 3) and limited the sample to 65 years of age or greater (Appendix Table 5 and Table 6).

Ethical approval was obtained from the Institutional Review Boards at Universidad Peruana Cayetano Heredia and Asociacion Benefica PRISMA in Peru, the Johns Hopkins Bloomberg School of Public Health in Baltimore, and the University of California, San Francisco.

## Results

In total, 373 participants were involved in the study of which approximately 24% had clinically significant depressive symptoms (Table 1). There were no differences in age, physical activity, hazardous alcohol consumption, BMI, site, prevalence of diabetes, hypertension diagnosis, current blood pressure medication use, heart disease diagnosis, current heart disease medication use or biomass fuel smoke exposure between the group with depressive symptoms and the group without depressive symptoms. However, there were statistically significant differences in sex, blood pressure, employment and education between the two groups. Females were more likely to have clinically significant depressive symptoms; 83.5% of participants with depressive symptoms were female while only 47.9% of participants without depressive symptoms were female. Participants with a primary education or less were more likely to have depressive symptoms, 56% (51/91) had depressive symptoms while 36.9% (104/282) did not. Of those participants that were unemployed, 46.2% (42/91) expressed depressive symptoms while 29.8% (84/282) did not. Finally, diastolic and systolic blood pressure were lower in the group that had clinically significant depressive symptoms.

**Table 1:** Characteristics of CRONICAS participants with and without depressive symptoms using CES-D.


	NO DEPRESSIVE SYMPTOMS (N = 282)	DEPRESSIVE SYMPTOMS (N = 91)	P VALUE

***Age (mean)***	56.2	58.3	0.15

***Female, n (%)***	135 (47.9)	76 (83.5)	<0.001

***Weekly physical activity, MET (mean)***	289.5	138.6	0.10

***Hazardous Alcohol Use, n (%)***	57 (20.2)	6 (6.6)	0.06

***Body mass index, kg/m*^2^ *(mean)***	27.7	27.7	0.99

***Pack-years of tobacco use (mean)***	1.2	0.4	0.15

***Primary education or less, n (%)***	104 (36.9)	51 (56.0)	0.001

***Pampas site, n (%)***	148 (52.5)	39 (42.9)	0.11

***Diastolic blood pressure, mmHg (mean)***	72.2	69.4	0.02

***Systolic blood pressure, mmHg (mean)***	114.3	109.3	0.01

***Hypertension diagnosis, n (%)***	39 (13.8)	14 (15.4)	0.71

***Current BP medication use, n (%)***	18 (6.4)	4 (4.4)	0.50

***Heart disease diagnosis, n (%)***	11 (3.9)	7 (7.7)	0.14

***Current heart disease medication use, n (%)***	10 (3.5)	4 (4.4)	0.70

***Diabetes, n (%)***	13 (4.6)	9(9.9)	0.06

***Biomass exposure, n (%)***	217 (77.0)	72 (79.1)	0.67

***Unemployed, n (%)***	84 (29.8)	42 (46.2)	0.004


In the minimally adjusted model, none of the echocardiographic measures were associated with clinically significant depressive symptoms except for septal e’ velocity, where there was a difference of –0.68 cm/s (95% CI –1.2 to –0.16, p = 0.01) in the depressive group compared with the non- depressive group. Left ventricular internal diameter at diastole, left ventricular internal diameter in systole, stroke volume, ejection fraction, left ventricular mass, left atrial diameter, E/A ratio and E/e’ ratio were not statistically different between the depressed group and non- depressed group. In the fully adjusted model, the septal e’ velocity remained statistically significant and again was the only echocardiographic measure to be different between individuals with and without depressive symptoms; there was a difference of –0.72 cm/s in the septal e’ velocity (95% CI –1.21 to –0.24, p = 0.004) between the depressive group compared with the non- depressive group.

**Figure 1:**
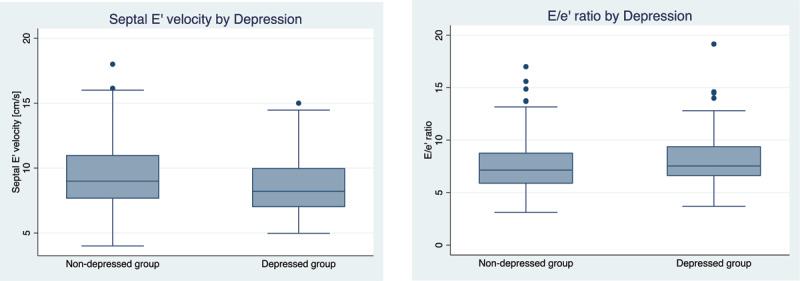
Septal e’ velocity and E/e’ ratio in depressed and non- depressed groups.

The average lateral e’ velocity was lower in the depressed group at 10.92 cm/s compared with the non-depressed group at 11.53 cm/s (Appendix: Table1). The average septal e’ velocity was also lower in the depressed group at 8.79 cm/s compared with the non-depressed group at 9.50 cm/s as was the median sepal e’ velocity (Figure 1). The average E/e’ was higher in the depressed group than the non-depressed group, 8.24 cm/s versus 7.57 cm/s respectively. The average percent of participants with an E/e’ greater than 12 is also higher in the depressed group at 5.6% compared to 4.1% in the non-depressed group.

In secondary analyses, when the CES-D score was treated as a continuous measure, there was an association with LV ejection fraction and LV mass in the minimally adjusted model that became statistically insignificant in the fully adjusted model. A one-point higher CES-D score was associated with a 0.30% lower LV mass (95% CI –0.01 to –3×10^–4^, p = 0.03), and a one-point higher CES-D score was associated with a 0.06% higher LV ejection fraction (95% CI 0.001 to 0.12, p = 0.05). Both of these measures became statistically insignificant in the fully adjusted model, however.

When limiting the sample to participants greater than or equal to 65 years of age, E/A ratio and E/e’ ratio was associated with depression. In the minimally adjusted model, the E/A ratio was 11.6% higher in the depressed group compared to the non-depressed group (95% CI –0.0001 to 0.22, p = 0.05). In the fully adjusted model there was no statistically significant association between E/A ratio and depression. In the fully adjusted model, there was a difference of 1.05 in the E/e’ ratio between the depressed and non-depressed group (95% CI –0.02 to 2.12, p = 0.05).

There was no association between clinically significant depressive participants and right heart structure or function. The right ventricular diameter at the base, mid- cavity and overall length was not statistically different between the groups in the minimally adjusted or the fully adjusted model. The right ventricular systolic pressure (RVSP) and the tricuspid valve systolic excursion were also not statistically different between the two groups (Table 3). In the secondary analysis when limiting the sample population to 65 years of age or greater, there was a 4.31mmHg difference in the right ventricular systolic pressure (RVSP) between the depressed group compared with the non-depressed group (95% CI 0.08 to 8.58, p = 0.05).

## Discussion

In a population-based sample of rural and urban Peruvians, we found an inverse association between the septal e’ velocity and clinically significant depressive symptoms after controlling for age, sex, site, biomass fuel use, height, BMI, total physical activity, prior diabetes diagnosis, pack-years of smoking, hazardous alcohol consumption and blood pressure. The e’ is a tissue Doppler signal that records the early movement of the mitral valve annulus in diastole and is used to assess diastolic function. In the setting of reduced myocardial compliance, velocity of the relaxation phase is slowed, resulting in reduced e’. This variable is incorporated into the E/e’ ratio (the ratio of the peak filling in early diastole relative to early diastolic annular velocity), commonly used to assess diastolic function. An elevated E/e’ ratio is associated with increased risk for incident heart failure in various longitudinal cohorts [14,15]. In this population, the depressed group had a numerical 0.47 increase in E/e’ ratio however it was not statistically significant (Table 2); nonetheless, it is worth pointing out the lower e’ velocity and higher trending E/e’ ratio in the clinically depressed group (Figure 1); whether a larger sample size would reveal more significant findings remains speculative.

**Table 2:** Multivariable linear regression analysis comparing left atrial and ventricular echocardiographic parameters in clinically significant depressive participants versus non- depressive participants. Multivariable model controls for age, gender, site, biomass utilization, height, BMI, total physical activity, prior diabetes diagnosis, pack- years of smoking, hazardous alcohol consumption as well as systolic and diastolic blood pressure.


	FULLY ADJUSTED β (95% CI)	P VALUE

***LV internal diameter, diastole, cm***	0.07 (–0.59, 0.20)	0.29

***LV internal diameter, systole, cm***	0.11 (–0.04, 0.27)	0.16

***LV stroke volume, mL***	0.41 (–3.14, 3.95)	0.82

***LV ejection fraction, %***	0.88 (–0.33, 2.09)	0.15

***LV mass, g (logged form)***	–0.04 (–0.10, 0.02)	0.22

***LA diameter, systole, cm***	–0.5 (–0.16, 0.07)	0.41

***LA area, four- chamber, cm* ^2^**	0.10 (–0.99, 1.19)	0.86

***LA area, two- chamber, cm* ^2^**	0.45 (–0.63, 1.53)	0.42

***E/A Ratio (logged form)***	0.003 (–0.06, 0.06)	0.93

***Lateral e’ Velocity, cm/s***	–0.27 (–0.76, 0.23)	0.29

***Septal e’ Velocity, cm/s***	**–0.72 (–1.21, –0.24)**	**0.004**

***E/e’ ratio***	0.47 (–0.08, 1.02)	0.09


**Table 3:** Multivariable linear regression analysis comparing right atrial and ventricular echocardiographic parameters in clinically significant depressive participants versus non- depressive participants. Multivariable model controls for age, gender, site, biomass utilization, height, BMI, total physical activity, prior diabetes diagnosis, pack- years of smoking, hazardous alcohol consumption as well as systolic and diastolic blood pressure.


	FULLY ADJUSTED β (95% CI)	P VALUE

***Right ventricular diameter, base, cm***	0.01 (–0.13, 0.15)	0.86

***Right ventricular diameter, mid- cavity, cm***	–0.004 (–0.14, 0.13)	0.95

***Right ventricular length, cm***	0.02 (–0.17, 0.21)	0.83

RVSP*, mm Hg	1.27 (–0.68, 3.23)	0.20

TAPSE*, cm	–0.06 (–0.15, 0.04)	0.26

***Right ventricular outflow tract time to peak velocity, ms***	0. 99 (–6.13, 8.10)	0.79


* RVSP, right ventricular systolic pressure; TAPSE, tricuspid annular plane systolic excursion.

**Figure 2:**
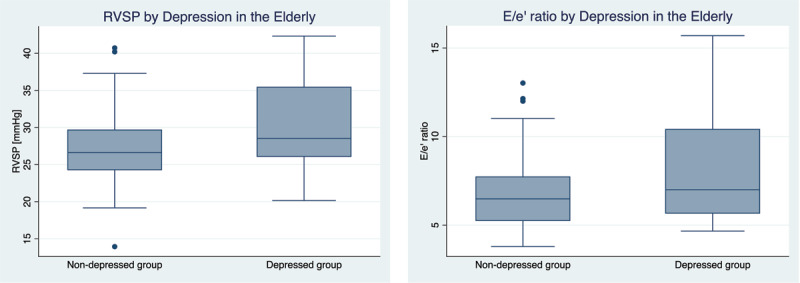
RVSP and E/e’ ratio in depressed and non- depressed groups greater than or equal to 65 years of age.

When conducting the secondary analysis in elderly participants, there was evidence of a higher E/e’ ratio (Appendix Table 5) and right ventricular systolic pressure (RVSP) (Appendix Table 6) in the depressed group that was statistically significant. Elevated RVSP is a component of diastolic dysfunction and may be associated with sub-clinical congestion. The decreased e’ velocity in conjunction with the elevated E/e’ ratio and RVSP (Figure 2) in the elderly sample might signify that there is a subclinical difference in diastolic function between the depressed group and non-depressed group. However, longitudinal follow-up of this cohort would be necessary to determine whether this observation in e’ velocity predicts cardiovascular outcomes including incident heart failure.

There is abundance of evidence that depression and heart failure are associated, however most studies examined effects of depression on patients with prevalent heart failure, where depression predicts heart failure progression and is associated with an elevated risk of cardiovascular disease mortality [16]. On the other hand, few studies examine whether depression is a contributing risk factor for the onset of heart failure and studies that do show conflicting results [4,5,17,18,19]. Abramson et al., William et al. and Wang et al. found an association between depression and heart failure but in patients with systolic hypertension, women and diabetic patients respectively [4,5,18]. Ogilvie et al. did not find any association between depression and incident heart failure in an elderly population [19]. Thus, our study provides an opportunity to probe pre-clinical manifestations of heart failure in an at-risk population, since the depressive symptom questionnaire preceded the collection of echocardiograms by three to four years. This study therefore provides useful information regarding the connection between depression and subsequent cardiac structure and function.

There are multiple potential mechanisms for how depression might impact diastolic function. Some studies show increased inflammatory markers in depression and heart failure such as C- reactive protein, TNF- alpha and IL- 6 [20,21]. Proinflammatory cytokines have direct effects on cardiac myocytes and promote cardiac myocyte hypertrophy which could lead to ventricular remodeling if there is no resolution of the inflammatory state [22]. Sympathetic nervous system and hypothalamic-pituitary axis activation could also result in increased vasoconstriction and afterload on the myocardium.

Our study is small in size, which limits the ability to extend our observation of lower e’ to other echocardiographic measures. There was an association between depression and E/A ratio, LV ejection fraction and LV mass in the minimally adjusted models that were not present in the fully adjusted model. This suggests the presence of residual confounding in the limited model. Given paucity of existing data against which to compare ours, we cannot state for certain a larger cohort would have been informative. Wang et al. is one of the few studies looking at the connection between depression and heart failure that used echocardiography to evaluate risk for incident disease. In their study, 274 asymptomatic diabetic elderly Australian participants were recruited with a mean follow- up time of 1.5 years. They found that poor diabetes control, left ventricular hypertrophy and depression all independently predicted incident heart failure [4]. Interestingly, they did not find associations between E/e’ ratio or e’ between the depressed and non- depressed group at the start of the study. Furthermore, E/e’ ratio was not independently predictive of incident HF in their study. The different results between our studies could potentially be due to unstable statistics due to small sample size and different measured population demographics. The Peruvian population in our study differed from the Australian study population because of the low prevalence of diabetes, tobacco use and hypertension. Also, the Peruvian population that we sampled was significantly younger than the Australian population and there was a higher prevalence of females with depression in our study. There is a well-known gender gap in depression incidence, women typically are twice as likely as males to have depression however in our depressed group 80% of those with depressive symptoms were female. This could be from cultural differences in expression of depression symptoms or small sample size of the depressed group.

Besides its small size, there were other limitations in the study design. Due to data availability, we were only able to conduct a cross-sectional study which can show associations but does not establish causality. A longitudinal design might have provided more information. In this study self-reported measures of depression symptoms were assessed with the CES-D survey and not collected by diagnostic psychiatric interviews. However, the CES-D has been validated as a measure for determining clinical depression in Spanish speaking countries [10]. One limitation for the depression variable is that we did not include the total duration of depressive symptoms or those who were taking medication for their depression. It is possible that there would be greater destructive effects on cardiac functioning for those suffering with depression for longer periods of time than those with newly diagnosed depressive symptoms and those not on medication: in theory short lived depression might not be associated with cardiac structure/function changes, while more severe, chronic depression might be. We performed analyses without correction for multiple comparisons; thus, risk for Type 1 error may be inflated. Lastly, in the depressed group 7.7% of the population had heart disease compared with 3.9% of the non-depressed group. Heart disease was not included in the fully adjusted model because there was no statistically significant difference between the two groups; however, given the higher proportion of heart disease in the depressed group this could be a possible cause for bias and could partially explain the differences in septal e’ velocity.

In conclusion, we found an association between clinically significant depressive symptoms and lower septal e’ velocity in this study of community dwelling Peruvians in Puno and Lima. There were no other obvious associations between clinically significant depressive symptoms and cardiac structure and function. Given the minimal number of studies looking at depression as a risk factor for cardiac dysfunction, more research is needed to further elucidate the connection between these two conditions.

## Data Accessibility Statement

The data that support the findings of this study are available from the corresponding author upon reasonable request.

## Additional File

The additional file for this article can be found as follows:

10.5334/gh.981.s1 Appendix. Tables 1 to 6.
