# Supplementary material for: Association Between Depressive Symptoms and Cardiac Structure and Function in a Peruvian Population
Source: Glob Heart. 2022 Oct 27;17(1):78. doi: 10.5334/gh.981 (PMC9615600; doi:10.5334/gh.981)
Supplement: Appendix. — Tables 1 to 6. [file gh-17-1-981-s1.pdf]

Appendix:

Table 1: Average left sided echocardiogram parameters in participants with and without depressive symptoms

	No Depressive symptoms mean (s.d.)	***Depressive symptoms mean (s.d.)***
LV internal diameter, diastole, cm	4.45 (0.60)	4.34 (0.56)
LV internal diameter, systole, cm	3.17 (0.67)	3.11 (0.70)
LV stroke volume, mL	76.20 (17.82)	70.82 (15.95)
LV ejection fraction, %	54.35 (5.99)	55.22 (5.22)
LV mass, g	179.68 (56.80)	155.93 (0.29)
LA diameter, systole, cm	3.56 (0.53)	3.43 (0.52)
LA diameter, four- chamber, cm^2	17.86 (4.21)	17.30 (4.51)
LA diameter, two- chamber, cm^2	19.64 (4.75)	19.21 (4.59)
E/A Ratio	1.04 (0.32)	1.03 (0.29)
Lateral e' Velocity, cm/s	11.53 (2.84)	10.92 (2.65)
Septal e' Velocity, cm/s	9.50 (2.68)	8.79 (2.27)
E/e'	7.57 (2.23)	8.24 (2.53)
% with E/e'>12	4.1%	5.6%

Table 2: Average right sided echocardiogram parameters in participants with and without depressive symptoms

	No Depressive symptoms mean (s.d.)	***Depressive symptoms mean (s.d.)***
Right ventricular diameter, base, cm	4.29 (0.59)	4.18 (0.61)
Right ventricular diameter, mid- cavity, cm	3.50 (0.59)	3.34 (0.53)
Right ventricular length, cm	7.59 (0.85)	7.40 (0.80)
RVSP*, mm Hg	27.11 (5.48)	27.85 (5.58)
TAPSE*, cm	2.32 (0.39)	2.24 (0.38)
Right ventricular outflow tract time to peak velocity, ms	187.67 (26.57)	187.69 (26.72)

*RVSP, right ventricular systolic pressure; TAPSE, tricuspid annular plane systolic excursion.

Table 3: Association between continuous CES-D scores and left atrial and ventricular echocardiographic parameters

	Fully Adjusted β (95% CI)	***P value***
LV internal diameter, diastole, cm	-6×10^{-4} (-0.01, 0.01)	0.85
LV internal diameter, systole, cm	0.001 (-0.01, 0.01)	0.70
LV stroke volume, mL	-0.03 (-0.19, 0.13)	0.71
LV ejection fraction, %	0.04 (-0.01, 0.10)	0.12
LV mass, g (logged form)	-0.002 (-0.01, 3×10^{-4})	0.10
LA diameter, systole, cm	7×10^{-4} (-0.005, 0.006)	0.81
LA area, four- chamber, cm2	0.01 (-0.04, 0.06)	0.62
LA area, two- chamber, cm2	0.03 (-0.03, 0.08)	0.36
E/A Ratio (logged form)	-7×10^{-5} (-0.003, 0.002)	0.96
Lateral e' Velocity, cm/s	0.005 (-0.02, 0.03)	0.70
Septal e' Velocity, cm/s	-0.004 (-0.03, 0.02)	0.75
E/e' ratio	-0.009 (-0.03, 0.01)	0.40

Table 4: Association between continuous CES-D scores and right atrial and ventricular echocardiographic parameters

	Fully Adjusted β (95% CI)	***P value***
Right ventricular diameter, base, cm	0.002 (-0.004, 0.009)	0.49
Right ventricular diameter, mid-cavity, cm	7×10^{-4} (-0.006, 0.01)	0.81
Right ventricular length, cm	0.002 (-0.01, 0.01)	0.73
RVSP*, mm Hg	0.004 (-0.09, 0.10)	0.94
TAPSE*, cm	6×10^{-4} (-0.004, 0.01)	0.81
Right ventricular outflow tract time to peak velocity, ms	0.22 (-0.13, 0.58)	0.22

*RVSP, right ventricular systolic pressure; TAPSE, tricuspid annular plane systolic excursion.

Table 5. Multivariable linear regression analysis comparing left atrial and ventricular echocardiographic parameters in clinically significant depressive participants versus non- depressive participants greater or equal to 65 years of age. Multivariable model controls for age, gender, site, biomass utilization, height, BMI, total physical activity, prior diabetes diagnosis, pack- years of smoking, hazardous alcohol consumption as well as systolic and diastolic blood pressure.

	Fully Adjusted β (95% CI)	***P value***
LV internal diameter, diastole, cm	0.22 (-0.05, 0.49)	0.11
LV internal diameter, systole, cm	0.14 (-0.17, 0.46)	0.37
LV stroke volume, mL	0.46 (-5.19, 6.11)	0.87
LV ejection fraction, %	2.18 (-0.18, 4.55)	0.07
LV mass, g (logged form)	-0.09 (-0.22, 0.04)	0.17
LA diameter, systole, cm	-0.14 (-0.20, 0.11)	0.27
LA area, four- chamber, cm^2	-0.25 (-2.38, 1.89)	0.82
LA area, two- chamber, cm^2	-0.46 (-2.79, 1.88)	0.70
E/A Ratio (logged form)	0.10 (-0.01, 0.20)	0.08
Lateral e' Velocity, cm/s	-0.42 (-1.17, 0.33)	0.27
Septal e' Velocity, cm/s	-0.49 (-1.22, 0.24)	0.18
E/e' ratio	**1.05 (-0.02, 2.12)**	**0.05**

Table 6. Multivariable linear regression analysis comparing right atrial and ventricular echocardiographic parameters in clinically significant depressive participants versus non- depressive participants greater or equal to 65 years of age. Multivariable model controls for age, gender, site, biomass utilization, height, BMI, total physical activity, prior diabetes diagnosis, pack- years of smoking, hazardous alcohol consumption as well as systolic and diastolic blood pressure.

	Fully Adjusted β (95% CI)	***P value***
Right ventricular diameter, base, cm	0.04 (-0.19, 0.27)	0.72
Right ventricular diameter, mid-cavity, cm	0.01 (-0.22, 0.24)	0.95
Right ventricular length, cm	-0.11 (-0.48, 0.25)	0.55
RVSP*, mm Hg	**4.31 (0.08, 8.53)**	**0.05**
TAPSE*, cm	-0.01 (-0.19, 0.17)	0.90
Right ventricular outflow tract time to peak velocity, ms	10.52 (-3.8, 24.83)	0.15

*RVSP, right ventricular systolic pressure; TAPSE, tricuspid annular plane systolic excursion.
